# Eye-Tracking Evidence That Verifiable Explanations Support Visual Evidence Checking in AI-Assisted Chest Radiograph Interpretation

**DOI:** 10.3390/jemr19030055

**Published:** 2026-05-15

**Authors:** Yong Han, Wumin Ouyang, Hemin Du, Mengyun Ma, Guanning Wang

**Affiliations:** 1 School of Design and Innovation, Shenzhen Technology University, Shenzhen 518000, China; u23092110161@cityu.edu.mo (Y.H.);; 2 Faculty of Innovation and Design, City University of Macau, Macau 999078, China

**Keywords:** eye tracking, verifiable explanations, visual evidence checking, medical AI interface, AI-assisted chest radiograph interpretation, human-factors evaluation

## Abstract

Evaluations of medical artificial intelligence (AI) explanations often rely on self-reported trust, perceived usefulness, acceptance, or final decision outcomes, while less directly characterizing whether users check evidence around AI outputs during decision making. In AI-assisted chest radiograph interpretation, a critical process-level question is whether users return from the AI output to the original image evidence when further scrutiny is needed. To address this question, we examined whether verifiable explanations—explanations designed to make AI recommendations checkable against the original image evidence—are associated with process markers of visual evidence checking in AI-assisted chest radiograph interpretation using eye-tracking and human-factors process measures. A 2 × 2 between-subjects experiment manipulated verifiable explanations (present vs. absent) and risk context (high vs. low), with AI recommendation correctness embedded at the trial level. Fifty-six clinically trained participants each completed 24 interpretation trials. Analyses focused primarily on gaze transitions between the AI output and the original image and dwell time on the original image, with response time and exploratory verification-related behavioral states used as auxiliary process measures. Verifiable explanations did not simply increase acceptance of AI recommendations. Instead, when AI recommendations were incorrect, they were most clearly associated with more frequent AI–image transitions and longer absolute dwell time on the original image evidence. Exploratory state-based analyses further suggested a lower tendency toward no-verify adopt under incorrect AI recommendations, but these findings were treated as complementary rather than primary evidence. Overall, the value of verifiable explanations lies not only in final decisions but in whether they make AI recommendations more inspectable against the original evidence. These findings provide eye-tracking evidence consistent with visual evidence checking in AI-assisted diagnostic interfaces and underscore the value of process-sensitive human-factors measures in medical AI evaluation.

## 1. Introduction

As artificial intelligence (AI) continues to enter medical image interpretation and clinical decision support, understanding users’ actual processing in AI-assisted tasks is becoming more important than focusing on endpoint outcomes alone [1,2,3]. In visually grounded tasks such as medical image interpretation, whether users ultimately adopt AI recommendations is insufficient to reveal how they process AI outputs, how they allocate attentional resources, or whether they genuinely return to the original evidence for further scrutiny [4]. By contrast, eye tracking can provide objective process evidence on attention allocation and decision making, and has therefore been increasingly used in medical human-factors research, decision support interface evaluation, and the identification of behavioral processes in high-risk tasks [5,6,7,8]. In such tasks, the value of eye tracking does not lie in replacing behavioral outcomes but in complementing the process-level information that conventional endpoint measures often fail to capture.

In research on medical AI explanations, evaluation has often been based on self-reported trust, perceived usefulness, acceptance, or final decision outcomes. However, these indicators cannot directly answer a more process-oriented question: when AI outputs warrant further scrutiny, do users actually return to the original evidence and show process patterns consistent with verification-oriented processing [9,10,11,12]? Recent studies have shown that, in medical AI decision support settings, relying solely on self-reports or endpoint behaviors can easily overlook key interaction processes between users and explanatory interfaces; eye-tracking measures, by contrast, can reveal more fine-grained relationships among explanatory information, original evidence, and final judgments [13,14,15]. At the same time, critical discussions of explainable AI have emphasized that explanations do not inherently lead to better judgments or safer use. If one cannot determine whether an explanation genuinely supports independent checking, then being “more explainable” in itself does not constitute a sufficient evaluative conclusion [16,17].

AI-assisted chest radiograph interpretation provides a methodologically advantageous setting for examining this process. On the one hand, chest radiograph interpretation relies heavily on sustained inspection of original image findings, and final judgments are difficult to separate from revisiting the underlying visual evidence. On the other hand, AI recommendations may often be useful but are not always correct, thereby creating a direct tension between AI outputs and original image evidence. In the present experiment, this tension was instantiated by making AI recommendations correct in 75% of trials and incorrect in 25% of trials, so that participants encountered an AI aid that was mostly reliable while still requiring evidence checking when advice was wrong. For this reason, chest radiograph interpretation is not only an application context for medical AI but also a task environment in which the process of visual evidence checking can be more clearly observed [13,18,19]. In this setting, if users remain primarily at the level of the AI conclusion and seldom return to the original image, their processing is closer to rapid acceptance. By contrast, if they frequently shift between the AI output and the original image, and allocate more attention to the original image, this pattern is more consistent with visual evidence checking and verification-oriented processing.

Building on this, the present study narrows its focus to a more specific form of explanation design: verifiable explanations. Here, verifiable explanations do not refer broadly to explanations that are simply “more detailed” or “more transparent”. Rather, they refer to whether the interface provides users with an entry point for returning to the original evidence and conducting further checking. In this sense, verifiable explanations are better understood as an interface affordance that supports evidence checking, rather than as a repackaging of the AI output itself [20,21,22]. Prior studies have shown that explanation type can affect clinical judgment, diagnostic performance, and trust-related outcomes, but this does not automatically mean that explanations genuinely support scrutiny of the original evidence [13,18,23]. Accordingly, the evaluation of verifiable explanations should not stop at whether users like, trust, or are more willing to accept AI recommendations. It should further examine whether such explanations are associated with changes in gaze transitions, image-directed attention, and verification-related behavioral states.

Against this background, this study focuses on visual evidence checking in AI-assisted chest radiograph interpretation, with particular attention to whether verifiable explanations are associated with process markers of visual evidence checking. Specifically, we address three interrelated questions. First, do verifiable explanations increase gaze transitions between the AI output and the original image, especially when AI recommendations are incorrect? Second, do they increase users’ attentional investment in the original image evidence, as reflected in longer dwell time on the image or a higher proportion of dwell time allocated to the image? Third, do these eye-tracking process measures correspond to exploratory verification-related behavioral states that provide behavioral context for visual evidence checking? By placing eye-tracking measures, temporal measures, and behavioral states within a unified analytic framework, this study aims to reveal, at the process level, whether explanatory interfaces support evidence checking and to provide more human-factors-grounded empirical evidence for the evaluation of medical AI interfaces.

## 2. Methods

### 2.1. Design and Overview

This study used a 2 × 2 between-subjects experimental design to examine whether verifiable explanations were associated with process markers of visual evidence checking during AI-assisted chest radiograph interpretation. The two between-subjects factors were verifiable explanation (present vs. absent) and risk context (high vs. low). AI recommendation correctness (correct vs. incorrect) was embedded at the trial level. Each participant completed 24 interpretation trials, consisting of 18 trials with correct AI recommendations and six trials with incorrect AI recommendations.

The 18:6 ratio was used to approximate a decision support setting in which AI advice is generally useful but imperfect. A balanced 50:50 ratio would have made AI errors unusually frequent and could have encouraged participants to treat the AI advice as unreliable by default. By contrast, the 75%/25% correct–incorrect ratio allowed participants to encounter a mostly reliable AI aid while still providing enough incorrect-AI trials to examine whether they revisited the original image evidence when the AI recommendation was wrong. The six incorrect-AI trials were distributed across three task blocks, with two incorrect recommendations embedded in each block, to reduce the likelihood that participants would infer a simple error pattern. Risk context was included as a secondary between-subjects factor to vary the perceived consequence pressure of the diagnostic judgment while keeping the image evidence and AI recommendation structure constant. Because the present research question centered on verifiable explanations and AI recommendation correctness, risk context was retained in the models as part of the experimental design rather than treated as the primary explanatory mechanism.

The present analysis treats eye-tracking and human-factors process measures as the primary outcomes. The analytic aim is not only to determine whether participants’ final decisions matched AI recommendations but also to examine how participants visually moved between the AI output and the original image evidence during decision making. Accordingly, all primary analyses were conducted at the trial level rather than after aggregation to participant-level means. This trial-level structure allowed repeated observations within participants to be modeled while preserving within-task variation in AI recommendation correctness, visual checking, and response behavior.

### 2.2. Participants, Ethics, and Group Allocation

This study was conducted in accordance with the Declaration of Helsinki and was approved by the Ethics Committee of Shenzhen Technology University (Serial No. SZTUIRP20260033) and the Ethics Committee of City University of Macau (Approval No. 202604081216). All participants provided written informed consent before the experiment and received fixed compensation upon completion. This study adhered to the principles of voluntary participation, the right to withdraw at any time, and anonymized data handling.

A total of 56 participants with clinical training backgrounds were included. Participants were assigned to one of four between-subjects conditions defined by explanation condition and risk context, with 14 participants in each cell. The sample included clinical clerkship medical students, resident physicians, and attending physicians. Because these subgroups may differ in chest radiograph interpretation experience, participant characteristics are reported by condition in Table 1. These characteristics included age, sex, clinical training level, years of clinical training or work experience, chest radiograph interpretation frequency, and self-rated chest radiograph familiarity. All participants completed the same AI-assisted chest radiograph interpretation task. In each trial, participants judged whether the current chest radiograph showed pleural effusion while receiving an AI recommendation. In the verifiable explanation condition, participants could open an evidence card related to the current AI recommendation through a View evidence entry on the interface; in the no-explanation condition, this entry was not displayed, while the remaining interface layout was kept consistent.

The theoretical total number of trials was 1344. After main analytic screening, 1343 trials were retained for behavior- and time-related analyses. Based on this set, trials with insufficient eye-tracking quality were further excluded, yielding 1284 trials for eye-tracking analyses.

### 2.3. Task, Case Selection, and AI Recommendation Construction

The experimental task was AI-assisted chest radiograph interpretation. Participants were required to judge whether the current chest radiograph showed pleural effusion while receiving an AI recommendation during the decision process. The task was restricted to a binary pleural effusion judgment to preserve clinical relevance while maintaining experimental control over AI recommendation correctness and verification-related behavior.

#### 2.3.1. Case Selection

Chest radiographs were selected from the publicly available NIH ChestX-ray14 data resource associated with the NIH chest X-ray benchmark publication by Wang et al. [24]. The task focused on pleural effusion because this finding is clinically relevant and visually grounded, making it suitable for examining whether participants returned to the original image evidence during AI-assisted interpretation.

Cases were screened according to two principles. First, selected images had to be sufficiently readable for the task, without severe display artifacts, missing key visual information, or abnormal presentation problems that would make the trial invalid. Second, cases were selected to avoid extreme difficulty levels. Images with overly obvious findings would reduce the task to simple error detection, whereas images with insufficient visual evidence would make the task closer to unsupported guessing. The final set consisted of 24 chest radiographs used in the formal task.

The reference label for constructing the experimental material was based on the pleural effusion label associated with the dataset record. Accordingly, AI recommendation correctness in this experiment should be understood as correctness relative to the reference label used for material construction, rather than as an independent clinical diagnosis made in a real care setting. This distinction is important because the present study was designed to examine visual checking behavior in an experimental human–AI decision task, not to validate a clinical diagnostic model.

#### 2.3.2. AI Recommendation Construction and Trial Order

AI recommendations were constructed as pre-specified AI advice for experimental control. The purpose was to create a controlled human–AI decision setting in which AI advice was generally useful but not perfectly reliable. Each participant completed 18 trials with correct AI recommendations and 6 trials with incorrect AI recommendations, corresponding to a 75%/25% correct–incorrect ratio. This ratio was intended to approximate a decision support system that is mostly reliable while still allowing this study to observe how participants responded when AI advice was wrong.

Correct AI recommendations matched the reference label used for constructing the trial. Incorrect AI recommendations were created by presenting the opposite recommendation for selected trials. These incorrect recommendations were designed to remain plausible enough to require checking rather than being immediately obvious as errors. In this sense, the incorrect-AI trials were intended to create a realistic need for visual evidence checking rather than a simple attention check situation.

The 24 trials were divided into three blocks of 8 trials, with 6 correct-AI trials and 2 incorrect-AI trials in each block. Within this structure, trial order was randomized across participants. Participants were not informed about the AI system’s overall accuracy, the correct–incorrect ratio, or the location of incorrect recommendations. The correctness assignment was fixed at the case level in the experimental materials; the same image was not presented with different AI-correctness labels across participants. Thus, trial order was randomized across participants, whereas the case-level correctness assignment was kept constant to preserve the intended 18:6 structure.

In the current experimental implementation, AI recommendation correctness was pre-assigned at the trial material level to maintain the intended 18:6 structure. Therefore, case-level difficulty was included as an adjustment variable in the statistical models. This design allowed this study to examine whether participants’ visual checking behavior differed when AI recommendations were correct versus incorrect, while acknowledging that correctness was experimentally embedded rather than produced by a deployed clinical AI system.

### 2.4. Interface Layout, Verifiable Explanation, and Risk Context Manipulation

**Figure 1:**
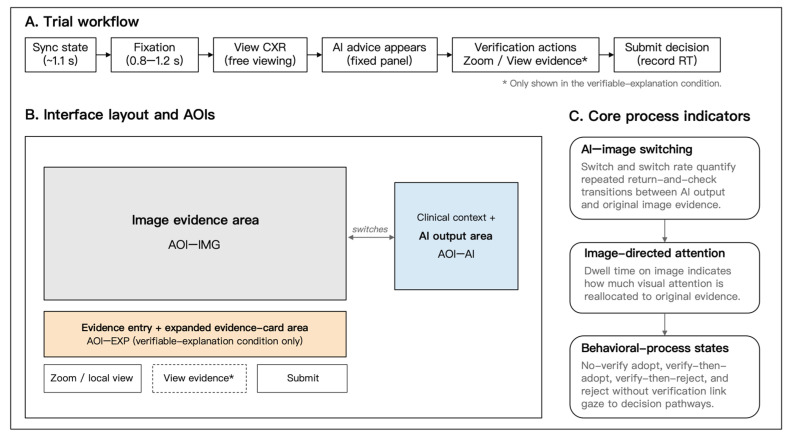
Schematic illustration of the study task flow, AOI layout, and core process indicators.

The experimental task was presented through a fixed-layout web interface. Across all trials, the original chest radiograph, AI output, task instruction, and submission button were displayed in consistent locations. This fixed layout was used to reduce variation in visual presentation and to support stable definition of interface regions for subsequent eye-tracking analyses. The overall task flow and spatial arrangement of the main interface components are illustrated in Figure 1. Additional schematic details of the no-explanation and verifiable explanation interfaces are provided in Supplementary Figure S1.

In the verifiable explanation condition, the interface included a View evidence entry. When participants clicked this entry, an evidence card was expanded in a fixed evidence-module area. The card provided brief verification cues linked to the current AI recommendation. These cues included a region to check on the chest radiograph and local visual feature cues relevant to pleural effusion, such as lower thoracic region inspection, blunting of the costophrenic angle, or fluid-related opacity. The evidence card was designed to direct participants back to the original image evidence for further inspection.

The evidence card did not provide an additional final diagnostic conclusion, ground truth, AI confidence score, heatmap, saliency map, or exemplar image. It also did not change the wording of the AI recommendation itself. Thus, the manipulation should be interpreted as an evidence card-based verifiable explanation interface: the explanation information was implemented through an evidence access affordance, but its purpose was to support checking of the original image evidence rather than to provide a substitute for participants’ own judgment.

In the no-explanation condition, the View evidence entry was not displayed, and participants could not open an evidence card. The remaining elements of the interface, including the original image region, AI output region, task instruction, and submission button, were kept consistent across explanation conditions. This arrangement was intended to isolate the presence of the verifiable explanation interface as much as possible while keeping the basic visual structure of the task stable.

Risk context was manipulated through the clinical background text associated with each trial. In the high-risk condition, the background emphasized that an incorrect judgment could delay timely follow-up examination or intervention. In the low-risk condition, the background framed the judgment as part of a routine review or follow-up context with lower short-term consequence pressure. The high- and low-risk texts were matched in presentation position and format and did not alter the chest radiograph, the AI recommendation, the evidence card structure, or the response options. Because no separate perceived-risk manipulation check was collected, risk context was treated as a secondary contextual factor varying consequence pressure without altering visual evidence or AI advice. Therefore, the risk context manipulation should be interpreted as an intended contextual framing of consequence pressure rather than as a confirmed difference in participants’ subjective risk perception.

### 2.5. Apparatus, AOIs, and Eye-Tracking Preprocessing

Eye-tracking data were collected using Tobii Pro Glasses 3 (Tobii AB, Danderyd, Sweden), a wearable eye-tracking system suitable for recording gaze behavior during screen-based interaction tasks. In the present experiment, gaze data were recorded at 100 Hz. The scene camera recorded video at 1920 × 1080 pixels and 25 frames per second, with a field of view of approximately 106° diagonal, 95° horizontal, and 63° vertical. A one-point calibration was performed for each participant before the formal task. Calibration quality was checked by visually confirming the alignment between gaze position and the calibration target, and calibration was repeated when tracking was judged unstable or visibly offset during the experiment.

The task was presented on a fixed-layout web interface. Three primary areas of interest (AOIs) were defined for the eye-tracking analyses: the original image evidence area (AOI-IMG), the AI output area (AOI-AI), and, in the verifiable explanation condition, the explanation-related evidence module area (AOI-EXP). AOI-IMG contained the chest radiograph presented for interpretation. AOI-AI contained the AI recommendation. AOI-EXP corresponded to the fixed region in which the evidence card could be displayed in the verifiable explanation condition. The fixed interface layout allowed these AOIs to be defined consistently across trials.

Eye-tracking data were processed in Tobii Pro Lab. Gaze data from the scene video were mapped onto the task interface plane, and AOIs were defined in the mapped screen space. A brief visual synchronization marker was presented before each trial to support alignment between the web event logs and the eye-tracking recording. Trial boundaries were then identified by combining the synchronization marker, trial ID, and event timestamps from the web interface.

Fixation-based AOI measures were exported from Tobii Pro Lab using the same project-level fixation-classification and AOI-mapping workflow across all participants and trials. The analysis relied on the fixation-based AOI metrics generated by Tobii Pro Lab and did not apply an additional user-defined fixation threshold or reclassify raw gaze samples outside the software. Because the present analyses focused on trial-level AOI measures rather than raw fixation parameter estimation, the exported fixation-based metrics were used to compute the core process indicators. For each trial, AI–image switches were computed as transitions between AOI-AI and AOI-IMG, and dwell time on image was computed as the total fixation duration within AOI-IMG. The explanation-related AOI was used to characterize interactions with the evidence module but was not itself treated as a primary eye-tracking outcome.

Eye-tracking quality control was conducted at the trial level. Missing gaze samples were not imputed at the analysis stage. A trial was marked as exclude_eye = 1 and excluded from eye-tracking analyses if the scene-to-interface mapping failed, if the trial boundary could not be reliably aligned with the behavioral log, if gaze data were largely missing during the decision interval, or if valid gaze samples were insufficient to compute the core AOI-based measures. Trials that met the behavioral inclusion criteria but failed these eye-tracking quality checks were retained for behavior- and response time-related analyses but excluded from eye-tracking models. Of the 1343 behavior-valid trials, 1284 trials were retained for eye-tracking analyses, corresponding to an eye-valid trial retention rate of 95.6%.

### 2.6. Measures

#### 2.6.1. Adoption and Auxiliary Performance Measures

Adopt indicated whether the participant’s final judgment was consistent with the AI recommendation in a given trial. It was coded as 1 when the participant’s final response matched the AI recommendation and 0 otherwise. This measure captured behavioral alignment with AI advice at the trial level, rather than subjective trust in the AI system.

Diagnostic accuracy was defined as whether the participant’s final judgment matched the reference label used for constructing the experimental material. In incorrect-AI trials, correct rejection was defined as a final judgment that rejected the AI recommendation and matched the reference label. These performance-related measures were used as auxiliary outcomes to contextualize the process findings, rather than as primary endpoints of the present analysis.

#### 2.6.2. Composite Verification Behavior

Verification-related behavior was recorded as a composite trial-level indicator. This indicator captured whether the participant initiated at least one predefined evidence-checking action during the trial, including image zooming or local inspection of the chest radiograph and, in the verifiable explanation condition, opening the evidence card. It was coded as 1 if at least one such action occurred during the trial and 0 otherwise.

Because evidence card opening was available only in the verifiable explanation condition, this composite Verify indicator was not treated as a fully symmetric measure of common verification behavior across explanation conditions. Instead, it was used as an auxiliary behavioral indicator for characterizing whether participants engaged with available evidence-checking functions during the trial. This distinction is important for interpreting the behavioral process states described below.

#### 2.6.3. Auxiliary Behavioral Process States

Behavioral process states were constructed by combining Adopt with the composite Verify indicator. Four states were defined: no-verify adopt, verify-then-adopt, verify-then-reject, and no-verify reject. No-verify adopt referred to trials in which the participant adopted the AI recommendation without initiating any recorded verification-related action. Verify-then-reject referred to trials in which the participant initiated a recorded verification-related action and then rejected the AI recommendation.

Because the composite Verify indicator included both common evidence-checking actions and, where available, evidence card opening, the state-based analyses were interpreted as auxiliary and exploratory behavioral evidence rather than as the primary basis for cross-condition inference. These analyses were retained to provide behavioral context for the eye-tracking findings, especially in trials with incorrect AI recommendations.

#### 2.6.4. AI–Image Switching Measures

AI–image switches referred to gaze transitions between the AI output area (AOI-AI) and the original image evidence area (AOI-IMG) within a trial. A switch was counted when gaze moved from AOI-AI to AOI-IMG or from AOI-IMG to AOI-AI. This measure was used as a process marker of back-and-forth visual comparison between the AI recommendation and the original image evidence.

Switch rate was defined as the number of AI–image switches divided by response time. Because the total number of switches can increase with longer decision time, Switch rate was included as a complementary measure of switching density per unit time.

#### 2.6.5. Image Dwell Measures

Dwell time on image referred to the total fixation duration within AOI-IMG during a trial. This measure captured the absolute amount of visual attention devoted to the original chest radiograph after the AI recommendation was presented.

Image dwell proportion was calculated as dwell time on image divided by response time. This measure reflected the relative proportion of the decision interval allocated to the original image evidence and was analyzed as a complementary attention allocation measure. It was interpreted separately from absolute dwell time because changes in total image dwell time do not necessarily imply changes in the relative allocation of decision time.

#### 2.6.6. Response Time and Case-Level Difficulty Index

Response time (RT) was defined as the time from the presentation of the AI recommendation to the participant’s final submission of the trial judgment. RT was used as an auxiliary temporal measure to characterize the decision time associated with evidence checking and final response selection.

Case-level difficulty was represented by an ordinal difficulty index recorded during trial material construction, with higher values indicating greater expected case difficulty. The index was assigned at the chest radiograph case level and inherited by all participant trials using the same case. It was included as a coarse adjustment variable in the statistical models to reduce the possibility that observed process differences were attributable solely to case-level variation, but it should not be interpreted as an independently validated psychometric measure of diagnostic difficulty.

### 2.7. Statistical Analysis

All analyses were conducted at the trial level. Behavior- and time-related analyses were based on the main analytic trials, whereas eye-tracking analyses were based on the eye-valid trials after eye-tracking quality screening. Participant ID was specified as the clustering unit to account for repeated observations from the same participant. Generalized estimating equation (GEE) models were used to estimate population-averaged effects under the repeated-trial data structure. GEE was selected because the inferential target was the population-averaged effects of the experimental factors across repeated trials, rather than participant-specific random-effect estimates. Models were specified with an independence working correlation structure and robust sandwich standard errors.

Exploratory behavioral process states were analyzed using logistic GEE models with a binomial family and logit link. Adopt, diagnostic accuracy, and correct rejection were used as auxiliary behavioral or performance-related outcomes to contextualize the process findings. Continuous or transformed process outcomes were analyzed using Gaussian GEE models with an identity link. The main eye-tracking models included verifiable explanation, AI recommendation correctness, risk context, case-level difficulty index, and the explanation × AI correctness interaction. Verifiable explanation, risk context, and AI correctness were dummy coded, with the no-explanation condition, low-risk condition, and incorrect-AI trials as the reference categories. The case-level difficulty index was entered as an ordinal adjustment variable.

Switch was modeled after log(count + 1) transformation to account for its count-like distribution and zero values. Dwell time on image was modeled after log(seconds) transformation to reduce right skewness. Switch rate was analyzed as a complementary switching density measure, defined as the number of AI–image switches divided by response time. Image dwell proportion was analyzed as a complementary relative attention allocation measure. Response time was used as an auxiliary temporal measure and to compute Switch rate and Image dwell proportion.

Exploratory behavioral process state models were estimated for no-verify adopt and verify-then-reject using the composite Verify indicator. Because this indicator included evidence card opening when available in the verifiable explanation condition, these models were interpreted as auxiliary state-based analyses rather than as the primary basis for cross-condition inference. Diagnostic accuracy and correct rejection were summarized as auxiliary performance-related measures to contextualize the process findings. In the binary task setting, correct rejection in incorrect-AI trials indicated that the participant rejected an incorrect AI recommendation and selected the response consistent with the reference label used for material construction.

Model-estimated marginal means and 95% confidence intervals were computed to aid interpretation of the GEE results. For log-transformed outcomes, estimates were back-transformed to the original scale where appropriate, allowing effect magnitudes to be interpreted in terms of switches per trial or seconds of image dwell time. Significance tests were two-sided, with α = 0.05. All statistical analyses were conducted in R. GEE models were fitted using the geepack package, estimated marginal means were computed using emmeans, and figures were generated using ggplot2. Participant clinical training level was reported descriptively by experimental condition and considered in interpreting the scope of the findings, but it was not included as a focal predictor in the primary models.

## 3. Results

### 3.1. AI–Image Switching as a Process Marker of Visual Evidence Checking

**Figure 2:**
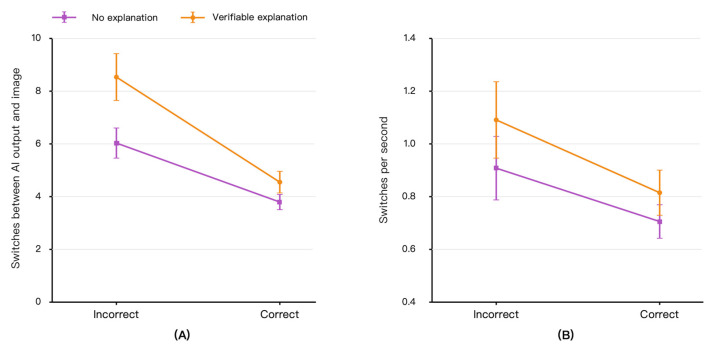
AI–image switching as a process marker of visual evidence checking. (**A**) Mean number of gaze transitions between the AI output area and the original image evidence area. (**B**) Switch rate, calculated as the number of AI–image switches divided by response time. Error bars indicate 95% confidence intervals.

Figure 2 presents gaze transitions between the AI output area and the original image evidence area. Overall, AI–image switching was higher when AI recommendations were incorrect than when they were correct, indicating that participants more frequently moved between the AI output and the original image when the AI advice warranted closer scrutiny. GEE analyses based on eye-valid trials showed a significant effect of AI recommendation correctness on Switch, after adjusting for risk context and case-level difficulty (β = −0.414, SE = 0.038, *p* < 0.001). Correct AI recommendations were associated with fewer AI–image switches than incorrect recommendations. The main effect of verifiable explanation was also significant (β = 0.311, SE = 0.065, *p* < 0.001), and the explanation × AI correctness interaction was significant (β = −0.155, SE = 0.054, *p* = 0.004; Table 2).

As shown in Figure 2A, the increase in AI–image switching was most evident when AI recommendations were incorrect. In incorrect-AI trials, participants in the no-explanation condition made an average of 6.04 AI–image switches per trial, whereas participants in the verifiable explanation condition made an average of 8.52 switches per trial. This corresponds to approximately 2.48 additional AI–image transitions per trial. This pattern suggests that verifiable explanations were associated with more frequent back-and-forth visual comparison between the AI recommendation and the original image evidence, particularly when the AI recommendation was unreliable.

Figure 2B shows Switch rate, defined as AI–image switches per unit response time. Switch rate showed a similar but more modest pattern. Verifiable explanation had a significant positive effect on Switch rate (β = 0.186, SE = 0.095, *p* = 0.049), and AI recommendation correctness had a significant negative effect (β = −0.196, SE = 0.042, *p* < 0.001). However, the explanation × AI correctness interaction was not significant for Switch rate (β = −0.079, SE = 0.067, *p* = 0.236). Descriptively, in incorrect-AI trials, Switch rate was 0.91 switches per second in the no-explanation condition and 1.09 switches per second in the verifiable explanation condition. Thus, Switch rate provides complementary evidence that the increase in switching was not merely a by-product of longer decision time, although the clearest evidence was observed for the total number of AI–image switches.

Taken together, Figure 2 and Table 2 indicate that AI–image switching was the clearest eye-tracking process marker in the present study. The results show that verifiable explanations were associated with more frequent visual transitions between the AI output and the original image evidence, especially in incorrect-AI trials. This pattern is consistent with increased visual evidence checking, while not implying that gaze transitions alone directly establish comprehension or diagnostic verification.

### 3.2. Visual Investment in the Original Image Evidence

**Figure 3:**
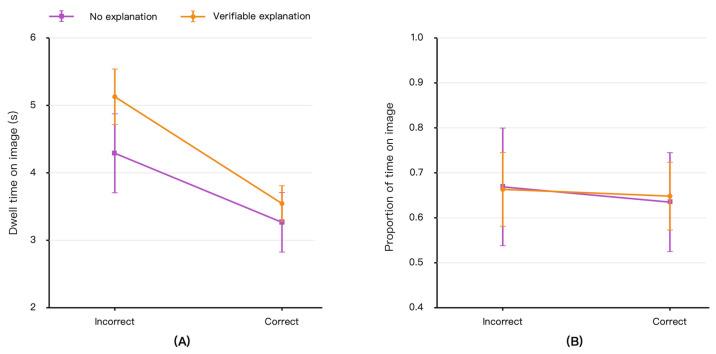
Visual investment in the original image evidence. (**A**) Mean dwell time on the original image evidence area. (**B**) Image dwell proportion, calculated as dwell time on image divided by response time. Error bars indicate 95% confidence intervals.

Figure 3 presents participants’ dwell time on the original image evidence and the proportion of decision time allocated to the image. Dwell time on image was greater when AI recommendations were incorrect than when they were correct, suggesting that participants spent more time inspecting the original chest radiograph when the AI recommendation required further scrutiny. GEE analyses showed a significant effect of AI recommendation correctness on dwell time on image (β = −0.269, SE = 0.024, *p* < 0.001), indicating shorter image dwell time in correct-AI trials than in incorrect-AI trials. Verifiable explanation also had a significant positive effect on dwell time on image (β = 0.215, SE = 0.073, *p* = 0.003), and the explanation × AI correctness interaction was significant (β = −0.094, SE = 0.037, *p* = 0.010; Table 2).

As shown in Figure 3A, verifiable explanations primarily increased absolute visual investment in the original image when AI recommendations were incorrect. In incorrect-AI trials, mean dwell time on the image was 4.26 s in the no-explanation condition and 5.12 s in the verifiable explanation condition, corresponding to approximately 0.86 additional seconds of image inspection per trial. This result supports the interpretation that verifiable explanations were associated not only with more frequent AI–image transitions but also with greater sustained inspection of the original image evidence when AI advice was unreliable.

Figure 3B shows Image dwell proportion, defined as dwell time on image divided by response time. Unlike absolute dwell time, Image dwell proportion showed weaker evidence of condition-related change. Neither the main effect of verifiable explanation (β = −0.003, SE = 0.076, *p* = 0.970) nor the explanation × AI correctness interaction (β = 0.015, SE = 0.033, *p* = 0.650) reached significance. AI recommendation correctness also did not show a significant effect on Image dwell proportion (β = −0.030, SE = 0.026, *p* = 0.253). Descriptively, in incorrect-AI trials, Image dwell proportion was similar in the no-explanation condition and the verifiable explanation condition.

Accordingly, the dwell time findings should be interpreted primarily as evidence of increased absolute image inspection time, rather than as strong evidence that verifiable explanations changed the relative allocation of decision time to the image. In other words, participants spent more time fixating on the original image evidence under verifiable explanations when AI advice was incorrect, but the proportion of the overall decision interval allocated to the image did not show a corresponding significant change.

### 3.3. Exploratory Behavioral Process States and Auxiliary Performance Context

To provide behavioral context for the eye-tracking findings, we examined exploratory behavioral process states constructed from Adopt and the composite Verify indicator. These states included no-verify adopt, verify-then-adopt, verify-then-reject, and no-verify reject. Because the composite Verify indicator included evidence card opening when available in the verifiable explanation condition, these state-based results were interpreted as auxiliary behavioral evidence rather than as fully symmetric cross-condition tests of common verification behavior.

**Figure 4:**
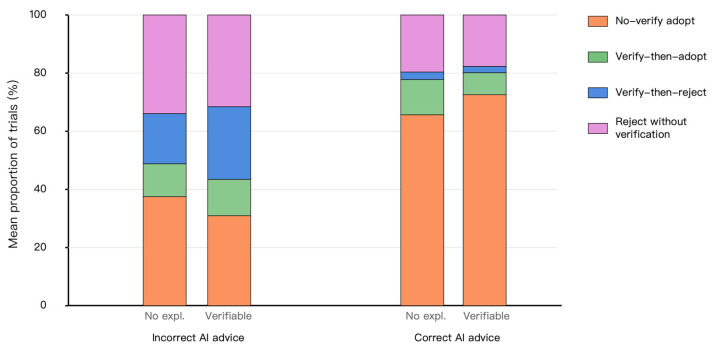
Exploratory behavioral process state patterns by AI recommendation correctness. The figure shows the proportional composition of behavioral process states separately for incorrect- and correct-AI trials. States were constructed using Adopt and the composite Verify indicator. Because the composite Verify indicator included evidence card opening when available in the verifiable explanation condition, these patterns should be interpreted as auxiliary behavioral context rather than as fully symmetric cross-condition tests of common verification behavior.

Figure 4 presents the proportional composition of these behavioral process states. In incorrect-AI trials, no-verify adopt was descriptively lower in the verifiable explanation condition than in the no-explanation condition. Specifically, no-verify adopt occurred in 37.5% of incorrect-AI trials in the no-explanation condition and 31.0% of incorrect-AI trials in the verifiable explanation condition. Conversely, verify-then-reject was descriptively higher in the verifiable explanation condition than in the no-explanation condition, occurring in 25.0% and 17.3% of incorrect-AI trials, respectively. These patterns are directionally consistent with the eye-tracking results, but they should be interpreted cautiously because they are based on the composite Verify indicator.

The exploratory binary GEE models are shown in Table 3. For no-verify adopt, AI recommendation correctness showed a significant positive effect (β = 1.170, SE = 0.178, *p* < 0.001), indicating that this state occurred more often when AI recommendations were correct. The explanation × AI correctness interaction was also significant (β = 0.610, SE = 0.281, *p* = 0.030), indicating that the association between verifiable explanations and no-verify adopt differed depending on whether the AI recommendation was correct or incorrect. In incorrect-AI trials, verifiable explanations were associated with a lower proportion of no-verify adopt, but this pattern was interpreted only as auxiliary behavioral context because of the composite and asymmetric nature of the Verify indicator.

For verify-then-reject, AI recommendation correctness showed a significant negative effect (β = −2.082, SE = 0.303, *p* < 0.001), indicating that this state occurred primarily when AI recommendations were incorrect. The coefficient for verifiable explanation in incorrect-AI trials was positive (β = 0.467, SE = 0.292), but this effect did not reach statistical significance (*p* = 0.110). The explanation × AI correctness interaction was also not significant (β = −0.630, SE = 0.440, *p* = 0.152). Therefore, the state-based evidence is stronger for a reduction in no-verify adopt than for an increase in verify-then-reject. The latter should be interpreted as directionally consistent but statistically weaker auxiliary evidence.

As an auxiliary performance context, diagnostic accuracy and correct rejection were also examined. In incorrect-AI trials, correct rejection was descriptively higher in the verifiable explanation condition than in the no-explanation condition (56.5% vs. 51.2%). This difference was not treated as evidence that verifiable explanations improved diagnostic performance. Rather, the performance-related results were used to contextualize the process-level findings and to indicate whether the observed visual checking patterns were accompanied by descriptively different response outcomes.

Overall, the exploratory state-based results are consistent with the main eye-tracking pattern: when AI recommendations were incorrect, verifiable explanations were associated with more AI–image switching, longer dwell time on the original image, and a lower tendency toward no-verify adopt. However, because the behavioral process states were based on a composite Verify indicator, they are best understood as auxiliary behavioral context rather than as the primary evidence for cross-condition differences in verification behavior.

## 4. Discussion

### 4.1. Main Findings

This study examined whether verifiable explanations were associated with process markers of visual evidence checking during AI-assisted chest radiograph interpretation. The clearest evidence came from AI–image switching. When AI recommendations were incorrect, participants showed more frequent gaze transitions between the AI output and the original image evidence, and this pattern was stronger in the verifiable explanation condition. Dwell time on the image provided a second source of process evidence: participants spent more time inspecting the original chest radiograph under verifiable explanations when AI advice was incorrect. By contrast, Image dwell proportion did not show corresponding significant changes, indicating that the dwell time effect should be interpreted primarily as increased absolute image inspection time rather than as a clear shift in relative attention allocation.

The exploratory behavioral process state analyses provided auxiliary behavioral context for these eye-tracking findings. In incorrect-AI trials, verifiable explanations were associated with a lower tendency toward no-verify adopt, whereas evidence for an increase in verify-then-reject was directionally consistent but statistically weaker. Correct rejection of incorrect AI recommendations was also descriptively higher in the verifiable explanation condition, but this performance-related pattern was treated only as contextual information rather than as evidence of improved diagnostic performance. Overall, the results suggest that verifiable explanations were most clearly associated with increased visual checking around unreliable AI advice, rather than with a general increase in AI-advice adoption. This pattern is consistent with recent eye-tracking work showing that safe and unsafe AI recommendations can be reflected not only in final responses but also in how clinicians visually revisit task-relevant evidence [13].

### 4.2. Process Interpretation of Visual Evidence Checking

The present findings support a process-level interpretation in which verifiable explanations functioned as an entry point back to the original image evidence. In the verifiable explanation condition, the evidence card did not provide an additional final diagnosis, ground truth, confidence score, heatmap, saliency map, or exemplar image. Instead, it provided cues that could direct participants to inspect relevant regions of the chest radiograph. The observed increase in AI–image switching therefore suggests that participants were more likely to establish a back-and-forth comparison between the AI recommendation and the original image evidence when the AI recommendation was unreliable. The corresponding increase in image dwell time further suggests greater sustained inspection of the original evidence.

This interpretation should remain bounded. Gaze transitions and dwell time do not directly demonstrate comprehension, diagnostic reasoning, or verification itself. More frequent switching should also not be interpreted as inherently beneficial, because it may reflect uncertainty, task difficulty, or increased interface demands in addition to evidence checking. Rather, they provide process-sensitive evidence about where visual attention was allocated and how participants moved between information sources during the decision interval. For this reason, the present study treats AI–image switching and image dwell time as process markers consistent with visual evidence checking, not as direct evidence of internal cognitive verification. This distinction is important for evaluating explanations in high-risk AI-assisted decision tasks: explanations should not be assumed to improve use simply because they are available or more detailed; their value depends on whether they support users in returning to task-relevant evidence when further scrutiny is needed [25,26]. From this perspective, the current findings extend prior work on explanation effects in chest radiograph interpretation by showing that explanation value can be examined not only through final diagnostic outcomes or trust-related responses, but also through observable visual checking processes [18].

### 4.3. Implications for Verifiable Explanation Design and Human-Factors Evaluation

These findings have implications for the design of explanation interfaces in medical AI. The evidence card-based interface used in this study is best understood as a verifiable explanation implemented through an evidence access affordance. Its purpose was not to provide a second answer or to make the AI output appear more authoritative, but to make the AI recommendation checkable against the original image evidence. This distinction is important because explanations in medical AI can have different effects depending on their content, format, and relationship to the underlying evidence [18,20,21,22,23]. In visually grounded tasks such as chest radiograph interpretation, an explanation may be more useful when it encourages users to inspect the original image than when it merely presents additional AI-generated information. This process-oriented view shifts the evaluation of medical AI explanations from asking whether an explanation is merely available or persuasive to asking whether it makes the AI recommendation inspectable against task-relevant evidence.

This study also illustrates the value of eye tracking as a human-factors method for evaluating AI-assisted diagnostic interfaces. Endpoint measures such as final adoption, accuracy, or subjective ratings remain important, but they may not reveal whether users actually inspect the evidence around an AI output. AI–image switching and image dwell time can provide process-sensitive indicators of how users distribute attention between the AI recommendation and the original evidence. This aligns with broader medical human-factors research that treats eye tracking as a tool for observing interface use, attentional allocation, and decision processes rather than merely as a visualization technique [5,6,7,8]. More broadly, eye-tracking research has shown that gaze measures can inform analyses of decision-related information processing and applied user experience in real-world settings [27,28]. Recent work has also begun to treat expert gaze as an indicator of medical AI interface usability and interaction quality [15]. In this context, the present study suggests that eye-tracking measures can complement behavioral outcomes by showing whether an explanation interface supports the process of visual evidence checking in a high-risk applied task.

### 4.4. Limitations and Future Research

Several limitations should be noted. First, this study was conducted in a controlled AI-assisted chest radiograph interpretation task focused on binary pleural effusion judgments. This setting was suitable for observing visual evidence checking because the original image remained central to the decision. However, the findings may not generalize directly to medical AI tasks with longer clinical workflows, multiple evidence sources, non-visual data, or evidence that cannot be revisited in a similarly direct manner.

Second, AI recommendation correctness was pre-assigned at the trial material level to create a controlled 18:6 correct–incorrect structure. Therefore, the findings should be interpreted as evidence from an experimental human–AI decision task rather than as validation of a deployed clinical AI model. Relatedly, the reference labels used to define AI correctness were based on the experimental material and should not be equated with independent clinical gold-standard diagnoses.

A related limitation is that AI recommendation correctness was not counterbalanced across cases or participants. The same chest radiograph was always paired with either a correct or an incorrect AI recommendation across participants. As a result, AI correctness effects cannot be fully separated from case-specific visual characteristics, such as image ambiguity, lesion conspicuity, or other image-level factors. Although the case-level difficulty index was included as an adjustment variable, this adjustment can only partially address material-level confounding and cannot substitute for a fully counterbalanced correctness design. Future studies should counterbalance AI correctness across cases or use larger case sets to model case identity more explicitly.

Third, the behavioral process state analyses were based on a composite Verify indicator. This indicator included common evidence-checking actions, such as image zooming or local inspection, and, in the verifiable explanation condition, opening the evidence card. Because evidence card opening was available only in the verifiable explanation condition, these state-based results should be interpreted as auxiliary and exploratory behavioral evidence rather than as fully symmetric cross-condition tests of common verification behavior. The available trial-level data did not retain sufficiently fine-grained action-specific event information to reconstruct a common action-only verification measure. Future work should record and analyze separate event log components, such as image zooming, local inspection, evidence card opening, time to first evidence-checking action, and gaze–click coupling.

Fourth, although this study included 56 clinically trained participants with balanced allocation across the four between-subjects conditions (14 participants per cell), this sample size may be insufficient for stable estimation of clinical training-level effects or training-level interactions. Future studies with larger and more stratified samples should examine how expertise shapes the use of verifiable explanations and the interpretation of AI advice. Finally, although the auxiliary performance results provided useful context, this study was not designed primarily to test whether verifiable explanations improve diagnostic accuracy. The evidence should therefore be interpreted as showing process-level changes in visual evidence checking, rather than as demonstrating improved clinical performance. Future research should examine whether these process markers are associated with decision quality in larger samples, more realistic clinical workflows, and longer-term human–AI interaction settings [29,30].

## 5. Conclusions

This study examined whether verifiable explanations are associated with process markers of visual evidence checking in AI-assisted chest radiograph interpretation. The findings indicate that the value of verifiable explanations lies not simply in making AI outputs more understandable or more acceptable, but in whether they help users return from AI recommendations to the original image evidence when further scrutiny is needed. In the present task, verifiable explanations were most clearly associated with more frequent AI–image switching and longer absolute dwell time on the original image when AI recommendations were incorrect. Exploratory behavioral process state analyses further suggested a lower tendency toward no-verify adopt under incorrect AI recommendations, although these state-based findings should be interpreted as auxiliary behavioral context rather than as primary evidence of cross-condition differences in verification behavior.

For high-risk medical AI tasks that depend strongly on visual evidence, the central design question for explanation interfaces is therefore not only whether they improve perceived clarity, acceptance, or final decision outcomes, but whether they make AI recommendations more inspectable against task-relevant evidence. The present findings suggest that eye-tracking and human-factors process measures can extend the evaluation of medical AI interfaces beyond endpoint outcomes by showing how users visually move between AI outputs and original evidence during decision making. At the same time, the evidence should be interpreted as process-level support for visual evidence checking rather than as direct proof of improved diagnostic performance or internal cognitive verification.

## Figures and Tables

**Table 1:** Participant characteristics and group allocation by experimental condition.

Characteristic	Overall	No Explanation/ Low-Risk	No Explanation/ High-Risk	Verifiable Explanation/ Low-Risk	Verifiable Explanation/ High-Risk
Sample size, *n*	56	14	14	14	14
Age, years	31.7 (4.9)	32.3 (3.6)	33.2 (4.0)	30.0 (6.5)	31.4 (5.2)
**Sex**					
Male	33 (58.9%)	9 (64.3%)	7 (50.0%)	8 (57.1%)	9 (64.3%)
Female	23 (41.1%)	5 (35.7%)	7 (50.0%)	6 (42.9%)	5 (35.7%)
**Training level**					
Clinical clerkship medical students	26 (46.4%)	6 (42.9%)	5 (35.7%)	5 (35.7%)	10 (71.4%)
Resident physicians	18 (32.1%)	3 (21.4%)	7 (50.0%)	6 (42.9%)	2 (14.3%)
Attending physicians	12 (21.4%)	5 (35.7%)	2 (14.3%)	3 (21.4%)	2 (14.3%)
Years of clinical training/work experience	4.8 (3.1)	5.5 (3.1)	4.5 (3.2)	5.0 (3.6)	4.0 (2.7)
**Chest radiograph interpretation frequency**					
Daily	14 (25.0%)	1 (7.1%)	4 (28.6%)	5 (35.7%)	4 (28.6%)
Several times per week	20 (35.7%)	6 (42.9%)	6 (42.9%)	3 (21.4%)	5 (35.7%)
Once per week	22 (39.3%)	7 (50.0%)	4 (28.6%)	6 (42.9%)	5 (35.7%)
Self-rated CXR familiarity (1–7)	4.5 (1.0)	4.4 (1.2)	4.4 (0.9)	4.5 (1.0)	4.7 (1.0)

Note. Values are presented as mean (SD) or *n* (%). CXR = chest radiograph.

**Table 2:** GEE models for eye-tracking process measures.

Predictor	Switch (Log[Count + 1]) β (SE)	*p*	Switch Rate β (SE)	*p*	Dwell on Image (Log s) β (SE)	*p*	Image Dwell Proportion β (SE)	*p*
Verifiable explanation	0.311 *** (0.065)	<0.001	0.186 * (0.095)	0.049	0.215 ** (0.073)	0.003	−0.003 (0.076)	0.970
AI correctness	−0.414 *** (0.038)	<0.001	−0.196 *** (0.042)	<0.001	−0.269 *** (0.024)	<0.001	−0.030 (0.026)	0.253
Risk context	−0.045 (0.053)	0.395	−0.076 (0.066)	0.247	0.064 (0.069)	0.352	0.013 (0.068)	0.853
Case-level difficulty	0.017 (0.017)	0.319	0.048 ** (0.017)	0.005	−0.005 (0.013)	0.709	0.010 (0.011)	0.345
Explanation × AI correctness	−0.155 ** (0.054)	0.004	−0.079 (0.067)	0.236	−0.094 * (0.037)	0.010	0.015 (0.033)	0.650

Note. GEE = generalized estimating equation; SE = standard error. Models were fitted using eye-valid trials, with participant as the clustering unit. No-explanation, low-risk, and incorrect-AI trials served as the reference categories. Switch was modeled after log(count + 1) transformation, and dwell time on image was modeled after log(seconds) transformation. Switch rate refers to AI–image switches divided by response time, and image dwell proportion refers to dwell time on image divided by response time. Robust sandwich standard errors were used. * *p* < 0.05, ** *p* < 0.01, *** *p* < 0.001.

**Table 3:** Exploratory binary GEE models for behavioral process states.

Predictor	No-Verify Adopt β (SE)	*p*	Verify-then-Reject β (SE)	*p*
Verifiable explanation	−0.288 (0.283)	0.309	0.467 (0.292)	0.110
AI correctness	1.170 *** (0.178)	<0.001	−2.082 *** (0.303)	<0.001
Risk context	−0.251 (0.183)	0.169	0.482 (0.321)	0.134
Case-level difficulty	0.055 (0.083)	0.508	−0.095 (0.169)	0.574
Explanation × AI correctness	0.610 * (0.281)	0.030	−0.630 (0.440)	0.152

Note. Results are from exploratory binary GEE models based on main analytic trials, with participant as the clustering unit. The state-based analyses were constructed using the composite Verify indicator and should be interpreted as auxiliary behavioral evidence. No-verify adopt was defined as Verify = 0 and Adopt = 1, whereas verify-then-reject was defined as Verify = 1 and Adopt = 0. No-explanation, low-risk, and incorrect-AI trials served as the reference categories. Robust sandwich standard errors were used. GEE = generalized estimating equation; SE = standard error. * *p* < 0.05, *** *p* < 0.001.

## Data Availability

The publicly available chest radiograph data resource used for constructing the experimental materials was the NIH ChestX-ray14 data release associated with the NIH chest X-ray benchmark publication by Wang et al. (2017) [24]. The study-generated trial-level eye-tracking, behavioral, and response time data are not publicly available due to privacy and ethical restrictions involving human participants. Aggregated descriptive values and model results needed to interpret the findings are reported in the manuscript; additional non-identifiable summary information may be made available from the corresponding author upon reasonable request, subject to ethical approval and institutional data governance requirements.

## Supplementary Materials

The following supporting information can be downloaded at https://www.mdpi.com/article/10.3390/jemr19030055/s1, Figure S1: Schematic comparison of the no-explanation and verifiable explanation conditions.

## Abbreviations

The following abbreviations are used in this manuscript:

AI	Artificial intelligence
AOI	Area of interest
GEE	Generalized estimating equation
RT	Response time
CXR	Chest radiograph
