# Supplementary material for: Eye-Tracking Evidence That Verifiable Explanations Support Visual Evidence Checking in AI-Assisted Chest Radiograph Interpretation
Source: J Eye Mov Res. 2026 May 15;19(3):55. doi: 10.3390/jemr19030055 (PMC13214853; doi:10.3390/jemr19030055)
Supplement: Supplementary file 1 [file jemr-19-00055-s001.zip › jemr-4279288-supplementary.pdf]

Supplementary Figure S1.

Schematic comparison of the no-explanation and verifiable-explanation conditions.

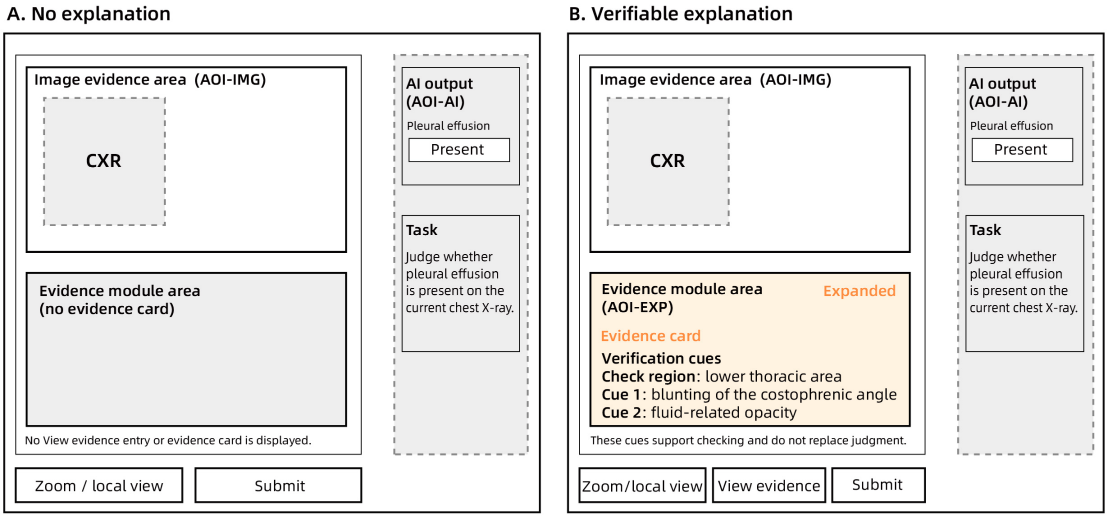

The schematic illustrates the interface components relevant to the explanation manipulation and AOI interpretation. Both conditions included the original image evidence area, AI output area, task instruction, zoom/local-view function, and submission button. In the no-explanation condition, the View evidence entry and evidence card were not displayed. In the verifiable-explanation condition, participants could open an evidence card linked to the current AI recommendation. The evidence card provided verification cues for checking the original chest radiograph but did not provide an additional final diagnosis, ground truth, AI confidence score, heatmap, saliency map, or exemplar image. CXR = chest radiograph.
